# Animal Models of ANCA Associated Vasculitis

**DOI:** 10.3389/fimmu.2020.00525

**Published:** 2020-04-09

**Authors:** Lani Shochet, Stephen Holdsworth, A. Richard Kitching

**Affiliations:** ^1^Centre for Inflammatory Diseases, Monash University Department of Medicine, Monash Medical Centre, Clayton, VIC, Australia; ^2^Department of Nephrology, Monash Health, Clayton, VIC, Australia; ^3^Department of Immunology, Monash Health, Clayton, VIC, Australia; ^4^Department of Pediatric Nephrology, Monash Health, Clayton, VIC, Australia

**Keywords:** autoantibodies, antineutrophil cytoplasmic, animal models, autoimmunity, glomerulonephritis, myeloperoxidase, proteinase 3, translational medical research

## Abstract

Anti-neutrophil cytoplasmic antibody (ANCA) associated vasculitis (AAV) is a rare and severe autoimmune multisystemic disease. Its pathogenesis involves multiple arms of the immune system, as well as complex interactions between immune cells and target organs. Experimental animal models of disease can provide the crucial link from human disease to translational research into new therapies. This is particularly true in AAV, due to low disease incidence and substantial disease heterogeneity. Animal models allow for controlled environments in which disease mechanisms can be defined, without the clinical confounders of environmental and lifestyle factors. To date, multiple animal models have been developed, each of which shed light on different disease pathways. Results from animal studies of AAV have played a crucial role in enhancing our understanding of disease mechanisms, and have provided direction toward newer targeted therapies. This review will summarize our understanding of AAV pathogenesis as has been gleaned from currently available animal models, as well as address their strengths and limitations. We will also discuss the potential for current and new animal models to further our understanding of this important condition.

## Introduction

The anti-neutrophil cytoplasmic antibody (ANCA) associated vasculitides (AAV) are autoimmune diseases characterized by systemic inflammation and subsequent destruction of small to medium blood vessels within target organs, particularly the kidneys and respiratory tract. It is a rare but life-threatening condition, with an incidence of 13–20 people per million per year worldwide, and a peak age of onset of 65–74 years (1). Syndromically, AAV can present as granulomatosis with polyangiitis (GPA; formerly known as Wegener's granulomatosis), microscopic polyangiitis (MPA) or eosinophilic granulomatosis with polyangiitis (EGPA). If untreated, mortality of AAV may be as high as 80% within 1 year of diagnosis (2). Treatment involves potent immunosuppressive agents that may have significant associated adverse effects, including infection and malignancy. Infection accounts for almost half of the deaths in treated patients in the first year (3). AAV-related glomerulonephritis (GN) is an important cause of end stage kidney disease and commonly defines outcomes in AAV.

AAV is a largely heterogeneous condition, with substantial variation in clinical presentation and sequelae. This variability presents significant challenges for patients and their doctors, as well as for recruitment and categorization in clinical studies. The hallmark of disease is the presence of auto-antibodies targeting proteins within azurophilic (primary) granules of neutrophils, with the two most clinically relevant autoantigens being proteinase 3 (PR3) and myeloperoxidase (MPO). These proteins are important players in the antimicrobial activity of neutrophils. AAV can be classified based on syndromic features or on the auto-antigen involved, specifically MPO-AAV or PR3-AAV. The Chapel Hill Consensus guidelines divide AAV into syndromic categories: GPA, MPA, and EGPA (4). The majority of people with GPA or MPA are ANCA positive at diagnosis, but in around 10% of patients ANCA are not detected in sera by conventional assays. Dual positive PR3-ANCA and MPO-ANCA serology is uncommon. Whilst most patients with GPA are PR3-ANCA positive, and similarly for MPA patients with MPO-ANCA, overlap between the clinical syndrome and ANCA specificity is incomplete. Observational studies have suggested that serological classification may better predict clinical features such as relapse rate (5), renal survival and mortality (6). The concept that PR3-AAV and MPO-AAV are different but related conditions is further supported by the identification of different genetic and epidemiological backgrounds between PR3-AAV and MPO-AAV [reviewed by Cornec et al. (7)].

**Figure 1:**
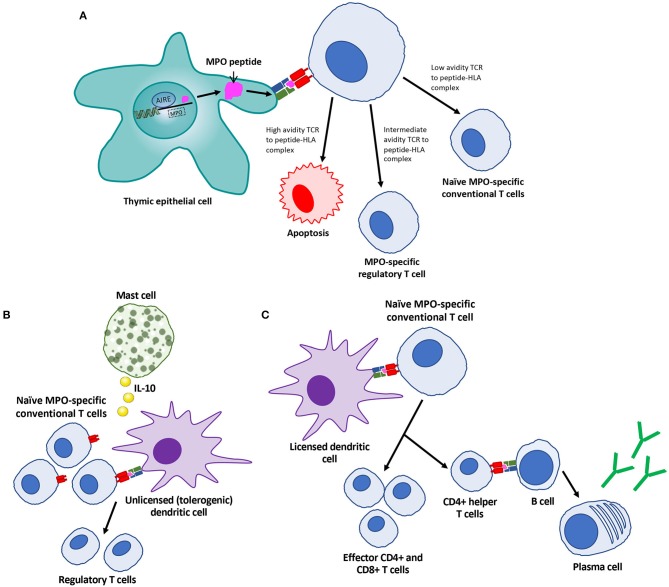
Features of central and peripheral mechanisms of tolerance to MPO as defined by animal models. **(A)** Central tolerance in the thymus is regulated by the transcription factor autoimmune regulator (AIRE) within the nucleus of thymic epithelial cells. This regulates autoantigen presentation to T cells on human leukocyte antigen (HLA) class II molecules, with subsequent T cell selection. **(B)** Peripherally, tolerance to MPO is maintained through MPO presentation on HLA class II by unlicensed dendritic cells to naïve T cells under the influence of IL-10 producing mast cells, promoting the development of regulatory T cells. **(C)** In certain situations, tolerance to MPO is lost, prompting expansion of T cells, and subsequent help for B cells to produce ANCA.

**Figure 2:**
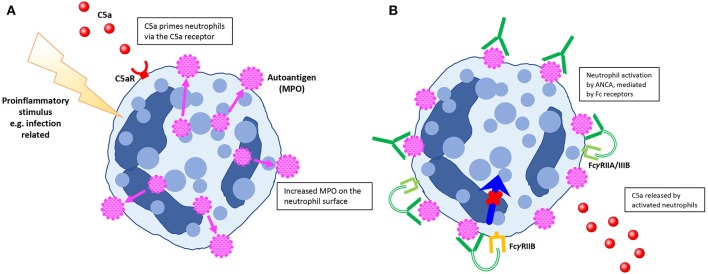
Features of neutrophil priming and activation by ANCA as defined by animal models. **(A)** Pro-inflammatory stimuli (including lipopolysaccharide and complement factor C5a) cause neutrophil priming, with increased expression of ANCA antigens on the neutrophil surface. **(B)** Mediated by regulatory Fcγ receptors, ANCA have the capacity to activate neutrophils. Neutrophil activation causes release of C5a, with subsequent complement pathway activation as well as further neutrophil priming.

**Figure 3:**
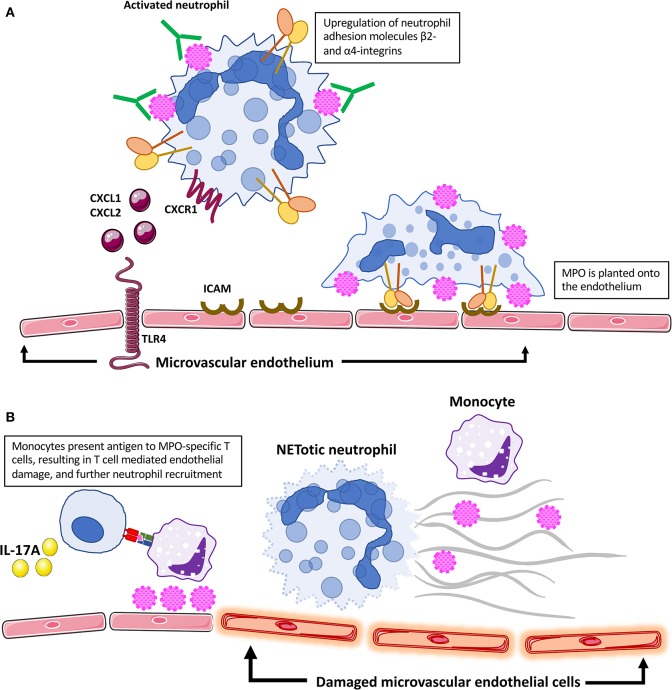
Features of neutrophil migration and adhesion, and endothelial damage as defined by animal models. **(A)** Activated neutrophils migrate to vulnerable vascular beds, including the glomerulus. The presence of TNF is associated with TLR4 upregulation on glomerular endothelial cells, which contributes to neutrophil migration through production of chemoattractants CXCL1 and CXCL2. After activation by ANCA, neutrophils express β2-integrins (LFA-1 and MAC-1), which enhance neutrophil adhesion to the glomerular endothelium. Neutrophil retention within the glomerular capillaries is moderated by the C5a receptor. **(B)** MPO is planted onto the glomerular endothelium, allowing local recognition by MPO-specific effector T cells and subsequent injury. Circulating monocytes have been shown experimentally to present antigens within glomeruli; however, microvascular endothelial cells and dendritic cells may also be involved in antigen recognition by effector T cells. Release of interleukin-17A (IL-17A) by T cells further encourages neutrophil migration. After localization to vulnerable vascular beds, neutrophils undergo necroptosis, and form neutrophil extracellular traps (NETs). This process promotes complement activation, and subsequent endothelial damage. Furthermore, NETs facilitate MPO presentation and propagation of the autoimmune response.

The pathophysiology of AAV is complex and remains incompletely understood. First, T and B cell tolerance to MPO or PR3 is lost, via mechanisms that remain incompletely described (Figure 1). Subsequently, with T cell help, autoreactive B cells or plasma cells produce ANCA. ANCA activate neutrophils (Figure 2) and induce their adherence to vulnerable microvascular beds, such as the glomerulus, where they degranulate and undergo NETosis, inducing endothelial injury (Figure 3). In this process, ANCA antigens are also deposited in the glomerulus, with the ability to be recognized by effector T cells, further contributing to injury [reviewed in Hutton et al. (8)]. It is hypothesized that monocyte/macrophages play a role later in disease (9); they themselves can be activated by ANCA and also have the capacity to present antigens to effector T cells (10).

Much of our understanding of the pathophysiology of AAV comes from animal models of disease, coupled with observations in human disease and *in vitro* studies. For example, since the discovery of ANCA in humans in 1982 (11), *in vitro* studies during the 1990s demonstrated that ANCA could activate human neutrophils (12–14), with animal studies later confirming the pathogenicity of ANCA *in vivo* with passive transfer of ANCA into mice (15). Similar advances have been made in understanding the role of effector T cells (16), complement (17), and the nature of T and B cell epitopes (18–20) in the pathogenesis of AAV. Furthermore, the judicious use of animal models has allowed pre-clinical investigation of new targeted therapies, exemplified by work on complement in a model involving the passive transfer of anti-MPO antibodies (21, 22). Whilst clinical and *in vitro* research into PR3-AAV is plentiful, no consistent PR3-AAV animal models currently exist, meaning that the *in vivo* understanding of AAV pathogenesis is based largely on models of anti-MPO disease. Whilst PR3-AAV and MPO-AAV share many pathological and clinical similarities, the differences between them span epidemiology, genetic predisposition, clinical features and histopathology [reviewed by Hilhorst et al. (23)]. Given these differences, it is important that animal models of PR3-AAV are developed, to further our understanding of the complexities of PR3-AAV, the differences between MPO-AAV and PR3-AAV, and to more accurately target treatments.

AAV is a unique autoimmune disease. Its pathogenesis involves all aspects of the immune system, with complex interplay between innate and adaptive immunity. It is one of only a few autoimmune diseases in which a single pathogenic autoantibody is measured. Furthermore, ANCA is pathogenic by binding to neutrophils and monocytes and inducing cellular activation, with resultant microvascular endothelial injury. However, depletion of the autoantibody alone may not be effective in disease control, and disease can be quiescent while the antibody remains detectable, suggesting redundancy in injurious autoimmune pathways.

Animal models of disease allow for a controlled environment, with the consequent ability to thoroughly interrogate human clinical observations and test hypotheses derived from these observations. AAV is a rare disease and consequently human studies often have limited numbers of patients. Patients are often heterogeneous and difficult to compare, due to confounders such as autoantigen specificity and potential epitope spreading throughout the course of disease, diverse clinical manifestations and immunosuppressive treatments. Animal models are also necessary for pre-clinical development of more effective, targeted treatments, before their translation into clinical experimentation. Ultimately though, models are just that: models. Whilst their use is invaluable in scientific research, they are only part of the puzzle of comprehensive understanding of a uniquely human disease.

This review will outline existing models which have contributed to the field of AAV. While many of these models have illuminated the biology of AAV, no single animal model presented here is able to replicate every stage of AAV, from loss of tolerance through to the development of end-organ fibrosis. Furthermore, these models still leave us with significant gaps in our disease understanding, including loss of tolerance, phenotypic heterogeneity and relapse prediction. Despite their critical roles in advancing our understanding of diseases, models have also been limited by their lack of consistency between laboratories, making research collaboration and conducting replication studies a significant challenge.

Of note, most animal models of AAV assess the impact of disease on the kidneys. Although renal disease is responsible for a major part of disease burden, other common organ manifestations are largely unstudied.

Different models have each shed light on different aspects of disease pathogenesis. Inspired by the identification of ANCA and its ability to activate neutrophils *in vitro*, earlier studies confirmed the pathogenicity of ANCA. The roles of priming and activating neutrophils, neutrophil migration to target organs and neutrophil degradation and extracellular trap formation have been investigated. The complex interplay between the adaptive and innate immune systems continues to be explored, including the role of T cells, complement and mast cells.

## Animal Models of MPO-AAV

In particular, animal models of MPO-AAV have been invaluable, in part due to significant homology with the human equivalent. The autoantigen itself is highly homologous, the pattern of ANCA binding to neutrophils is similar and the effects in the kidney are comparable, with a pauci-immune focal segmental crescentic GN. Table 1 summarizes selected models of MPO-ANCA associated renal vasculitis.

**Table 1:** Selected models of MPO-ANCA associated renal vasculitis.

	**Animal**	**Severity (+ to +++)^a^**	**Duration (effector phase)**	**Contribution to knowledge of pathogenesis^b^**	**Limitations**
**EXPERIMENTAL MPO-AAV**					
**Passive transfer**					No model studies active autoimmunity to MPO
Anti-MPO serum with anti-GBM Ab (24)	Wistar rats	++ to +++	3 or 15 h, or 14 days	Neutrophil activation is a prerequisite	Dual hit required Strong linear IgG deposition
Transfer of anti-MPO Ab (15)	C57BL/6 or *Rag2^−/^*^−^ mice	++	6 days	Proof of pathogenic role of anti-MPO Ab and neutrophils	Not strictly autoimmune (anti-MPO Ab raised in *Mpo^−/−^* mice)
Transfer of splenocytes from MPO-immunized *Mpo*^−/−^ mice (15)	*Rag2^−/^*^−^ mice	++	13 days	Injury mediated by MPO specific cells	Some immune complex deposition Not strictly autoimmune Immunodeficient recipients
Transfer of MPO intact bone marrow to MPO-immunized *Mpo^−/^*^−^ mice (25)	*Mpo^−/^*^−^ mice	++	8 weeks	MPO expression by leukocytes is required for anti-MPO Ab effects	Not strictly autoimmune in the induction of immunity Requires bone marrow transplantation
Transfer of effector MPO-specific CD4+(18, 26) or CD8+ (20) T cells/T cell clones	*Rag1^−/^*^−^ mice	++	14 days	MPO-specific CD4+/CD8+ T cells recognize MPO planted in the glomerulus, then effect injury	Anti-MPO Ab have been used for antigen deposition/recognition, but often uses sheep anti-mouse GBM Immunodeficient recipients
**Active autoimmunity**					
Active autoimmunity, with disease trigger: neutrophil lysosomal enzyme extract with H_2_O_2_ (27), ischemia/reperfusion (28), low-dose anti-GBM Ab (29)	Brown Norway rats	++ to +++	10 days	MPO-ANCA alone may not be sufficient for disease; trigger required	Significant IgG and C3 deposition Some versions technically challenging
Active autoimmunity in GN-susceptible rats (30)	WKY rats	++	6 weeks	Loss of tolerance to MPO after immunization	Rat strain specific No clear demarcation between induction of immunity and effector responses
Active autoimmunity, with disease trigger (16)	C57BL/6 mice	+ to ++	4–5 days	Understanding of steps in antigen recognition and role of T cells as effectors	Requires trigger Short term effector phase due to the development of active immune responses to foreign globulin

a *+, mild; ++, moderate; +++ severe*.

b *Only initial contribution listed due to space limitations*.

*Ab, antibody; ANCA, anti-neutrophil cytoplasmic antibodies; GBM, glomerular basement membrane; GN, glomerulonephritis; MPO, myeloperoxidase; Rag, recombination activating gene; WKY, Wistar Kyoto*.

### Passive Transfer of Anti-MPO Antibodies, Splenocytes, or MPO-Specific T Cells

#### Transfer of Anti-MPO Antibodies

Models centered on passive transfer of MPO-ANCA-like antibodies have been used in several laboratories to elucidate the complex pathogenesis of disease (15, 25, 31). Antibodies are usually generated by immunizing MPO deficient mice. While these antibodies are similar to MPO-ANCA, they are generated in mice that are not tolerant to MPO (15, 32) and therefore are most accurately described as anti-MPO antibodies. Models based on these principles have been valuable in explaining several areas of the effector response in AAV. These include the role of ANCA, neutrophil priming, activation, migration and adhesion, followed by endothelial injury. Furthermore, targets for potential treatments have been identified and trialed (21).

ANCA were initially identified in patients with segmental necrotising GN in the 1980s (11). While subsequent *in vitro* studies showed that ANCA can cause neutrophil activation and degranulation (12, 14, 33, 34), the first *in vivo* animal model data supporting the pathogenicity of ANCA were not published until many years later, when Kobayashi et al. showed enhancement of glomerular injury caused by anti-glomerular basement membrane (GBM) antibodies when co-administered with anti-MPO serum (24). Mouse models of anti-MPO GN involving passive transfer of anti-MPO antibodies were subsequently developed. Transfer of anti-MPO IgG, from MPO-immunized *Mpo*^−/−^ mice, into C57BL/6 mice and *Rag2*^−/−^ mice (that lack T and B lymphocytes) caused focal necrotising and crescentic pauci-immune GN, demonstrating the pathogenicity of ANCA *in vivo* and its role in acute glomerular injury (15). A key role for neutrophils as effectors in this model was demonstrated by neutrophil depletion that completely protected mice from glomerular histological injury, suggesting that ANCA induced glomerular injury was neutrophil mediated and that neutrophils were a major ANCA target (35).

Whilst passive transfer of anti-MPO antibodies without neutrophil priming has been shown to cause GN (15), disease is usually more severe when pro-inflammatory signals, such as lipopolysaccharide (LPS), are administered around the time of antibody transfer. Administration of LPS shortly after anti-MPO antibody transfer, with subsequent elevation in tumor necrosis factor (TNF) and circulating MPO levels, was shown to result in a significantly greater proportion of glomerular crescents and glomerular necrosis. This effect could be attenuated by TNF blockade (32). Neutrophil numbers, in addition to priming, were shown to be important in later experiments. Daily granulocyte colony stimulating factor (G-CSF) injections, causing an increase in circulating neutrophils, were administered in addition to LPS in the passive transfer model, leading to more severe disease (31). Based on *in vitro* experiments, the ability for ANCA to activate neutrophils is thought to be dependent on Fcγ receptors, in particular FcγRIIA (36–38). *In vivo* data supports a regulatory role for FcγRIIB in ANCA mediated injury, with *Fcgr2b*^−/−^ mice pre-treated with LPS and anti-MPO antibodies developing increased glomerular injury compared with FcγRIIB intact mice (39).

Given the role of complement as a potent mediator in vascular inflammation in other diseases, Xiao et al. hypothesized that despite the paucity of complement deposition in renal biopsies in AAV, complement activation may well be an important player. Xiao et al. showed *in vitro* that complement is activated after stimulation of human neutrophils with ANCA, via C3a detection (17), with subsequent experiments confirming that this effect was through activation of the C5a receptor (C5aR) (40). C5 deficient mice or mice treated with a neutralizing anti-C5a antibody were protected from glomerular damage after passive transfer of anti-MPO IgG (41). In addition to neutrophil priming, activation of the C5aR on dendritic cells promotes autoimmunity to MPO (22). A C5aR antagonist CCX168 (avacopan) protected mice expressing human C5aR from glomerular injury (21), with subsequent translation into human clinical trials of this compound in the treatment of AAV (42). The membrane attack complex does not seem to play a significant role, showing that C5a's effects are via the C5aR (21).

Further work has explored the relative contributions of other complement factors. C3 depletion with Cobra Venom Factor (CVF) prevented GN after passive transfer of anti-MPO IgG or anti-MPO splenocytes (17), though C3aR deficient mice are not protected from glomerular injury after passive transfer of anti-MPO IgG (43), and C3aR is not required for neutrophil priming (40). As such, it is conceivable that since C3 is upstream in the complement cascade, its role in neutrophil activation is as a precursor for C5a generation.

The alternative pathway of complement appears to be the dominant pathway in neutrophil activation in experimental AAV. Deficiency of factor B, which is specifically involved in alternate pathway activation, protected mice from development of disease. In contrast, mice deficient in C4, required for activation of the classical and lectin pathways, were not protected (44). Initiation of complement activation appears to be independent of both properdin, which is released by activated neutrophils and can initiate the alternative pathway, and MBL-associated serine protease 2 (MASP-2), which can activate the lectin pathway (41). Properdin deficient mice were not protected from disease after anti-MPO antibody transfer. As such, the initiator of the alternative pathway in AAV remains unknown. Recent *in vitro* data suggests that NETs may provide a scaffold for complement activation (45), including allowing MPO interaction with Factor H, an important alternative complement pathway regulator (46).

After activation by ANCA, neutrophils are attracted to vulnerable vascular beds, where they degranulate and subsequently cause endothelial damage and disease. Inhibiting neutrophil migration may serve as a promising target for treatment of AAV. Experiments by Summers et al. explored the drivers of neutrophil recruitment to target organs (47). Highly purified LPS, which specifically engages toll-like receptor 4 (TLR4), was associated with increased neutrophil recruitment and functional renal injury when injected with anti-MPO antibodies. TLR4 was predominantly upregulated in glomerular endothelial cells in mice and human cell lines, leading to production of functionally important neutrophil chemoattractants. These effects were mediated by TLR4 expressed both by immune cells and by endothelial cells secreting CXCL1 and CXCL2, the murine homologs of CXCL8 interleukin-8 (IL-8). Subsequent studies in human MPO-ANCA associated GN demonstrated that intrarenal TLR4 expression correlates with the extent of renal injury (48).

Intravital microscopy in both rats and mice after passive transfer of anti-MPO antibodies has enabled a more detailed exploration of interactions between neutrophils and the vascular endothelium. Initial work in this area studied the rat mesenteric post capillary venule (49). CXCL1 was applied topically to the rat mesentery, and rat MPO-ANCA or control antibodies were injected. Topical CXCL1 alone significantly increased endothelial leukocyte adhesion, as well as transmigration that was further enhanced in rats receiving MPO-ANCA after CXCL1. Similar findings were demonstrated in an active immunization model. In both models, changes were not seen in rats with MPO-ANCA that did not receive CXCL1, highlighting the requirement for chemokine involvement in endothelial injury. Nolan et al. imaged post capillary venules in the cremaster muscle to assess the response to administration of anti-MPO IgG after pre-treatment with several different cytokines (50). Within minutes of anti-MPO IgG administration, there was reduced leukocyte rolling, enhanced adhesion and increased transmigration across the endothelium. These interactions were Fcγ receptor and β2 integrin dependent, and were mediated by cytokines, in particular TNF. Neutrophil migration and adhesion is partly dependent on activation of the kinin system, with genetic deficiency or pharmacological blockade of bradykinin receptor 1 associated with reduced neutrophil surface expression of adhesion molecules and attenuated GN (51).

Post capillary venules are the primary sites of physiological and pathological leukocyte recruitment, but in AAV leukocytes are recruited to capillary beds, particularly glomerular capillaries. The glomerular microvasculature is not only an important target in AAV, but also is unique in the manner by which leukocytes interact with the endothelium (52, 53); as such, the effects of anti-MPO antibodies in promoting leukocyte adhesion within the glomerulus itself were examined. In a murine model, anti-MPO antibodies were shown to bind to circulating neutrophils, altering adhesion molecules and inducing glomerular leukocyte adhesion via multiple pathways. Mechanisms of adhesion to the glomerular endothelium were affected by the dose of anti-MPO antibodies, as well as pre-treatment with LPS to model a pre-existing inflammatory state (53). In the presence of LPS, low dose anti-MPO antibodies induced CD11a/CD18 dependent glomerular neutrophil adhesion, while higher dose antibodies induced α4 integrin dependent adhesion. In these experiments, the same stimuli did not induce leukocyte recruitment to cremasteric post capillary venules, consistent with clinical observations of preferential and often selective renal involvement of glomeruli in AAV. *In vivo* multiphoton microscopy, with the capacity to image glomerular leukocyte behaviors over time without the risk of endothelial photoactivation, has demonstrated that neutrophils are retained in glomeruli in inflammation, are activated and crawl bidirectionally within the glomerular microvasculature. A single activated neutrophil can crawl at 10 μm per minute, a finding that may explain the acute segmental glomerular necrotic lesions seen in AAV (52). This system has also furthered the understanding of the role of complement in neutrophil migration and endothelial injury. C5a was shown to play an important role in MPO-ANCA induced neutrophil retention and activation within the glomerulus (22).

After activation by ANCA and localization to vulnerable vascular beds, neutrophils degranulate but they can also undergo cell death via multiple mechanisms, including neutrophil extracellular trap (NET) formation (known as NETosis) (54). These web-like histone containing structures contain MPO and PR3, and many pro-inflammatory proteins and peptides, some of which are endogenous TLR agonists (55). NETs are released within the microvasulature, where they not only contribute to endothelial injury but also may activate TLRs expressed on resident tissue cells (48, 56) and may promote local alternate complement pathway activation (57). They also deposit the autoantigens MPO and PR3 in target tissues, making them potentially able to be presented to effector antigen-specific T cells (58). While mechanistic understanding of NETs has largely been gleaned from *in vitro* studies (54, 59), the functional role of NETs as effectors of injury has also been translated to animal models of AAV. After anti-MPO antibody transfer into mice, enhanced degradation of NETs by administration of DNase I was protective against development of anti-MPO antibody GN (45).

Endothelial cell activation and injury in AAV is mediated by signal transduction pathways, including NF-κB signaling. Activation of NF-κB by external stimuli allows for migration of NF-κB into the nucleus, to promote transcription of pro-inflammatory signals. Choi et al. identified that release of TNF from ANCA-activated neutrophils upregulated NF-κB in endothelial cells, with subsequent IL-8 production. Prophylactic application of immunoliposomes which downregulated endothelial NF-κB was associated with reduced glomerular necrosis (60).

Whilst the role of neutrophils is well-established, monocyte/macrophages have context driven pro-inflammatory roles in immune diseases and play a pathogenic role in AAV. In addition to their general pro-inflammatory properties, primed monocytes also express MPO and PR3 on the cell surface and so plausibly may also play a role in disease (10, 61, 62). Anti-MPO antibody transfer experiments showed that selective monocyte depletion limited histological but not functional kidney injury (9). Further evidence of the potential role of macrophages in AAV is gleaned from clinical data. Macrophages comprise a significant proportion of leukocytes in kidneys of people with AAV, especially in early disease (63–65). They generate macrophage extracellular traps (METs) and ~25% of CD68+ macrophages are positive for MPO protein by immunostaining (55). Furthermore elevated urinary soluble CD163, shed by monocytes and macrophages, is strongly associated with active renal vasculitis and has potential as a biomarker to detect renal relapses of AAV (66).

The demonstration of the pathogenicity of ANCA in this model has prompted strategies to alter interactions between ANCA and effector leukocytes. Modification of ANCA IgG glycosylation via IgG hydrolysis limited the clinical and pathological features of GN (67). Interrupting leukocyte signaling has been examined in this and other models (68). A specific inhibitor of p38 mitogen-activated protein kinase (MAPK) administered either before or after transfer of anti-MPO antibodies limited glomerular crescent formation without reducing haematuria or proteinuria (69). Dooley et al. trialed EDO-S101, a drug combining the alkylating agent bendamustine with the histone deacetylase inhibitor, vorinostat. Whilst pretreatment with this drug reduced circulating leukocytes, it did not prevent development of GN in the passive transfer model (70).

The effects of factors such as environmental exposures on AAV are unclear. However, age is a risk factor for AAV development and severity (6). To evaluate the effect of MPO-ANCA in aged animals, anti-MPO antibodies were passively transferred into recipient aged mice. Aged mice developed more severe GN, with increased circulating and glomerular neutrophils and increased gene expression of pro-inflammatory cytokines (71). Although costly, aged mice may better model aged humans and in the future may contribute significantly to the understanding of disease pathogenesis and to studies of new treatments for AAV. Similarly, mice exposed to infections may have an immune system more analogous to adult humans (72, 73), and although these systems currently come with several drawbacks, they may contribute to our understanding in the future.

#### Transfer of Splenocytes From MPO-Immunized Mice

As well as transfer of antibodies, splenocytes from MPO-immunized *Mpo*^−/−^ mice can induce nephritis. Transfer of splenocytes (including both T and B cells) from *Mpo*^−/−^ mice hyperimmunized with either MPO (or BSA as a control protein) into immunodeficient *Rag2*^−/−^ mice resulted in detectable serum anti-MPO antibodies within 3 days with dose-dependent necrotising and crescentic GN and renal impairment by day 13, as well as variable involvement of other organs. There was significant glomerular immune complex deposition both in mice receiving anti-MPO splenocytes and control mice which received anti-BSA splenocytes (15).

#### Transfer of MPO Intact Bone Marrow to MPO-Immunized Mpo^-/-^ Mice

The pathogenicity of anti-MPO antibodies and the requirement for MPO expression by innate leukocytes was further explored in a bone marrow transfer model where *Mpo*^−/−^ mice were immunized with MPO, irradiated and reconstituted with bone marrow from MPO intact mice (25). Mice that received wild type bone marrow developed GN with crescent formation in ~30% of glomeruli. MPO-immunized mice that received *Mpo*^−/−^ bone marrow remained disease free. Disease could be induced by transfer of anti-MPO antibodies into non-immunized *Mpo*^−/−^ mice reconstituted with bone marrow from MPO-intact mice. Collectively, this model confirms the requirement for MPO to be present on leukocytes, most likely neutrophils. Further evidence of the pathogenicity of ANCA was provided in this model by Bontscho et al., with a reduction in renal disease, fewer glomerular neutrophils and lower anti-MPO antibody titres after administration of the proteasome inhibitor bortezomib (or corticosteroids and cyclophosphamide) post bone marrow transplantation (74).

This model has also been used to enhance our understanding of the pathways involved in anti-MPO antibody-neutrophil interactions and injury. One such pathway involves phosphoinositide 3-kinase (PI3K), which controls neutrophil respiratory burst and migration. Bone marrow cells from PI3Kγ-deficient (but MPO intact) mice resulted in only mild glomerular abnormalities. Further, a small molecule inhibitor of PI3Kγ (AS605250) protected mice from development of GN after transfer of wild type bone marrow (68). Neutrophil serine proteases (NSPs: cathepsin G, neutrophil elastase and proteinase 3) mediate inflammation and injury. To determine their roles, bone marrow transfer studies were undertaken. Mice receiving NSP deficient bone marrow or marrow from mice lacking dipeptidyl peptidase I (DPPI, required for activation of NSPs) were protected from developing crescentic GN, possibly due to disrupted signaling via IL-1β (75). In addition to the passive anti-MPO antibody studies outlined above, this bone marrow transplant model has helped established a role for C5a-C5aR interactions using C5aR deficient mice (40), as well as a role for NETs in the effector phase of MPO-AAV using Receptor-interacting protein kinase-3 (RIPK3) deficient mice (45).

#### Transfer of Effector CD4+ or CD8+ T Cells

Passive T cell transfer studies have defined a role for CD4+ and CD8+ cells in AAV. Autoreactive CD4+ and CD8+ cells are present in people with AAV (76–79). The obligatory and well-documented presence of the autoantigen in inflamed tissues in these conditions (due to local myeloid cell release of MPO and PR3) (55) implies a role for T cells as effectors of injury, assuming these antigens can be processed and presented to antigen-specific T cells locally. Model antigens have been shown to recruit T cells to glomeruli to cause injury (80), and at least for MPO, a similar process operates. Experimentally, transfer of antigen-specific CD4+ or CD8+ T cells (as clones) induces necrotising GN when MPO is planted in glomeruli (18, 20).

Experimentally, MPO or MPO peptides can be deposited in glomeruli in several ways. Early experiments perfused renal arteries with MPO (27). In the context of T cell transfer studies, MPO has been deposited in glomeruli in three ways. Firstly, injection of low dose heterologous anti-GBM antibodies induces transient neutrophil recruitment with MPO deposition in glomeruli. Secondly passive transfer of anti-MPO antibodies with LPS results in similar deposition of MPO (18). Thirdly, immunogenic MPO peptides can be coupled to a “carrier” monoclonal mouse anti-GBM monoclonal IgG1 antibody (18, 81). The nephritogenicity of the antibody itself is negligible as mouse IgG1 does not fix complement and has low affinity for Fc receptors. The transfer of clones specific for MPO, either effector Th1 CD4+ T cells or CD8+ cells, to *Rag1*^−/−^ mice results in severe necrotising GN after MPO is planted in glomeruli. Glomerular injury is mild to minimal after transfer of cells specific to an irrelevant specificity (ovalbumin) (18, 20). Antigen can be presented intravascularly by patrolling monocytes (58) and may also be presented locally by glomerular endothelial cells, while later in disease dendritic cells infiltrate diseased glomeruli in human AAV (82).

### Experimental Autoimmune Anti-MPO Disease Induced by Active Immunization With MPO

Whilst passive transfer experiments have contributed greatly to our understanding of effector mechanisms in AAV, these systems do not include loss of tolerance to MPO (or PR3). In most passive transfer studies, recipients receive fixed doses of antibodies or cells from donor MPO-immunized animals. Thus, tolerance is not broken in recipients and they do not develop active autoimmunity, meaning that fundamental questions relating to loss of tolerance and potential therapies to re-establish tolerance cannot be addressed. As such, active immunization models of disease have been developed in both rats and mice.

#### Active Experimental Anti-MPO Disease in Rats

Immunization of Brown Norway rats with MPO, with subsequent development of MPO-ANCA, is not sufficient for development of disease. However, necrotising crescentic GN in the presence of ANCA has been initiated by causing glomerular endothelial damage either through perfusion with lysosomal extract containing MPO (27), ischemia/reperfusion (28), or a sub-nephritogenic dose of anti-GBM antibodies (also known as “nephrotoxic serum”) (29). There are several possible explanations for the increased injury observed by these additional triggers. However, subsequent studies have demonstrated that this enhanced injury is largely due to planting of MPO, as an antigen, within glomerular capillaries and subsequent recognition by effector T cells (16, 18, 20). This occurs either by direct localization of MPO (or antigenic MPO peptides), or by transient recruitment of neutrophils that deposit MPO, allowing local recognition of MPO by MPO-specific effector T cells. A variation of these models in rats investigated the role of products released from activated neutrophils, including MPO, in the pathogenicity of MPO-ANCA, especially in the development of ANCA-associated pulmonary disease (83, 84).

Active MPO immunization of the GN-susceptible Wistar Kyoto (WKY) rat strain results in loss of tolerance to MPO with ANCA, mild GN and at times pulmonary disease (30). The role of TNF has been explored in this model. Treatment with anti-TNF antibodies 4 weeks after immunization significantly curtailed active AAV, both functionally and pathologically, without affecting MPO-ANCA titres (85). However, administration of TNF did not enhance disease and TNF levels were not different in rats immunized with MPO compared with controls (86). Unfortunately, a human trial of anti-TNF therapy using etanercept were not successful (87), despite anecdotal reporting of its benefit in humans (88). Although not effective in a mouse passive transfer model of disease, treatment with EDO-S101, that combines an alkylating agent with a histone deacetylase inhibitor, limited renal and lung pathology in MPO-immunized WKY rats even when given after disease establishment, suggesting significant effects on active anti-MPO autoimmunity (70). Co-administration of a sub-nephritogenic dose of anti-GBM antibody to WKY rats enhances glomerular crescent formation and albuminuria. This is associated with overexpression of glomerular chemoattractants including CXCL1 and CXCL2, and enhanced neutrophil activation and adherence to endothelial cells (86).

#### Active Anti-MPO Glomerulonephritis in Mice

Based on studies in rats in the 1990s (29), an active model of AAV was developed in mice (16). In these models, autoimmunity to MPO is initiated in genetically intact mice, but the MPO-ANCA that develops is not sufficient in itself to induce disease. GN is triggered by injection of a low dose of sheep anti-mouse glomerular basement membrane (anti-GBM) antibodies that, as in the T cell transfer models described above, transiently recruits neutrophils to glomeruli (89) with deposition of MPO. When mice are immunized with MPO, moderate injury develops, but immunization with an irrelevant antigen (usually ovalbumin) results in minimal injury mediated only by the anti-GBM antibodies. Initial studies in this model demonstrated that it is dependent on MPO, as *Mpo*^−/−^ mice did not develop disease despite mounting an immune response to MPO. This outcome confirms that the use of low-dose anti-GBM antibodies can achieve neutrophil influx and MPO deposition with minimal potential confounding injury. CD4+ T cell depletion in the effector phase markedly attenuated injury, demonstrating the role of effector CD4+ T cells. Furthermore, B cell deficient mice developed similar disease to mice with intact B cells thereby proving the antibody-independent role of T cells in this model of anti-MPO associated GN. Later experiments further highlighted the role of T-cell mediated injury. GN caused by passive transfer of anti-MPO antibodies into B cell deficient mice was enhanced by pre-immunization with MPO to induce MPO-specific CD4+ T cells. These effects could be prevented with T cell depletion (26). Effectively, this active model demonstrated that in the presence of MPO locally in human AAV, most likely via ANCA-activated neutrophil adhesion, effector T cells mediate injury. In the model, ANCA is bypassed by the use of low dose anti-GBM antibodies to deposit MPO in glomeruli, resulting in an effector response that is akin to a delayed type hypersensitivity reaction.

This model has led to further investigation into the role of T cells in AAV. CD4+ effector T cells, in particular upon differentiation to Th17 cells, mediate production of neutrophil chemoattractants by tissue cells via release of IL-17A (80). After MPO immunization, IL-17A deficient mice were protected from disease, via effects on both neutrophils and macrophages (90). Studies using mice deficient in Th1 or Th17 defining cytokines have shown an initial Th17 dominant lesion followed later by a Th1 dominant outcome, where Th17 defining cytokines were redundant (81). Other types of T cells have also been implicated, including CD8+ T cells (20). Unconventional γδ T cells, also a source of IL-17A, play a role in glomerular T cell and neutrophil recruitment. Mice genetically deficient in γδ T cells developed less severe disease compared to wild type mice (91).

The role of FcγRIIB beyond its involvement in neutrophil activation by ANCA was assessed in this T cell dependent model of disease. Whilst not expressed on T cells, FcγRIIB was shown to inhibit T cell responses via a tonic inhibitory effect on professional antigen presenting cells, with FcγRIIB deficient mice having increased CD4+ T cells, macrophage and neutrophil recruitment to glomeruli, resulting in increased glomerular injury (39). In addition to its effects on T and B cell responses, FcγRIIB is also likely to directly limit the activity of effector macrophages. In an analogous manner, complement plays an important role in disease beyond neutrophil stimulation. C5aR1 modulates development of autoimmunity to MPO, with *C5ar1*^−/−^ mice relatively protected from T cell mediated disease, as dendritic cells lacking the C5aR1 are not able to fully activate anti-MPO T cells (22). In contrast, the absence of C3aR did not affect the development of disease in this active model (43).

Given the induction of autoimmunity in this model, it can be used to interrogate mechanisms of loss of tolerance to MPO. Tan et al. determined that the transcription factor autoimmune regulator (AIRE) promotes the expression of MPO in the thymus, enabling the deletion of autoreactive anti-MPO T cells in the thymus (Figure 1A). This work also found that depletion of regulatory T cells led to more anti-MPO-specific T cells, higher ANCA titres and more severe GN (92). Mast cells contribute to peripheral tolerance to MPO, through IL-10 mediated effects on regulatory T cells (Figure 1B) (93). Furthermore, autoimmunity and GN can be attenuated by disodium cromoglicate, a mast cell stabilizer (94).

Animal models have been used to discover and define immunodominant MPO T and B cell epitopes. Specifically, three papers have collectively defined an MPO epitope hot spot in a similar region of the MPO heavy chain, with pathogenic CD4+ T cells (mouse studies across several MHC II allomorphs) (18), B cells (human studies with functional murine models) (95) and CD8+ T cells (a pathogenic MPO peptide in mice that is likely to also bind to common HLA Class I alleles) (20). The CD4+ T cell and B cell epitopes have been validated in human studies (96). As antigen-specific tolerogenic therapies have potential as curative therapies in autoimmune disease (97, 98), knowledge of these epitopes has been used in combination with several tolerogenic platforms in AAV, including nasal tolerance (99), injection of MPO peptide loaded apoptotic cells (81), and injection of tolerogenic dendritic cells (100). While the exact type of regulatory cell varies with different strategies, the mechanism of action in each of these three studies is via the generation of MPO-specific T cells that regulate and suppress established anti-MPO autoimmunity. Collectively these studies show proof of concept that using the previous defined immunodominant MPO T cell epitope can be used in tolerogenic studies, with antigen-specific effects.

Clinical observations have suggested a correlation between infection and AAV (101, 102). There are several potential mechanistic explanations for this association that are not mutually exclusive. One potential explanation involves the engagement of TLRs, expressed on leukocytes or on intrinsic tissue cells, which stimulate immunity and can alter the strength and direction of the immune response. When mice were co-immunized with MPO and TLR ligand, Summers et al. observed enhanced cellular and humoral autoimmunity, compared with mice immunized with MPO alone (103). TLR2 ligands directed Th17 anti-MPO autoimmunity while TLR9 ligands supported Th1 immunity. Infection may also be associated with loss of tolerance to MPO through molecular mimicry. Immunization of mice with a plasmid-encoded peptide with some sequence homology with the T cell immunodominant MPO epitope, but found only in some strains of *Staphylococcus aureus*, induced anti-MPO autoimmunity and vasculitis (104).

A variation of models that immunize with MPO was published by Yumura et al. (105) BSA administration induced anti-BSA antibodies, which themselves activate neutrophils causing release of MPO, promoting loss of tolerance to MPO and the development of anti-MPO antibodies. Mice developed features of pulmonary disease and severe crescentic GN. Despite pulmonary disease being a common clinical feature in AAV, most animal models are limited to renal manifestations of AAV.

## Animal Models of PR3-AAV

Compared with experimental models of MPO-AAV, consistent animal models of PR3-AAV (summarized in Table 2) have been difficult to establish. There are several possible reasons for this. Compared with MPO, human and mouse PR3 have limited homology of 68%, and the antigenic determinants of human PR3 may not be preserved in mouse PR3 (106). Peripheral blood neutrophil numbers are lower in mice (107) and total white blood cell numbers may be lower compared to humans, depending on mouse strain (108). In contrast to humans, where neutrophils account for up to 70% of total leukocytes, in commonly used inbred mouse strains (C57BL/6 and 129/Sv) neutrophils account for 7.7 and 14% of total white blood cells (9–9.4 × 10^3^/μL), respectively (109). Under resting conditions, PR3 is expressed on the plasma membrane in humans through its co-expression with CD177, via a hydrophobic patch on PR3 (110). Murine PR3 lacks this hydrophobic patch, possibly accounting for why PR3 is not strongly expressed on the mouse neutrophil surface, especially in the resting state (111). This may be significant in the development of murine models of PR3-AAV, as it is believed that expression of PR3 on the neutrophil surface is critical to the pathogenesis of AAV (112). Furthermore, *in vitro* evidence suggests that PR3-ANCA causes neutrophil activation via FcγRIIA expressed on human neutrophils (37). Importantly, this Fc receptor does not have a murine ortholog (113), which may contribute to the inability of PR3-ANCA to activate mouse neutrophils.

**Table 2:** Selected models of ANCA associated vasculitis (see Table 1 for animal models of MPO-ANCA associated renal vasculitis).

	**Animal**	**Severity (+ to +++)^a^**	**Duration (effector phase)**	**Contribution to knowledge of pathogenesis^b^**	**Limitations**
**EXPERIMENTAL PR3-AAV**					
**Passive transfer**					
PR3-specific splenocyte transfer (118)	NOD-SCID mice	++ to +++	20-40 days	Anti-PR3 B and T cells mediate injury; role for regulatory immune response	Poor homology between human and mouse PR3
Passive transfer of human PR3-ANCA to mice reconstituted with human stem cells (119)	Irradiated NOD-SCID-*Ill2rγ^−/−^* mice	+	6 days	*In vivo* evidence for human PR3-ANCA pathogenicity	Human PR3 present on chimeric neutrophils and monocytes required Challenging immune reconstitution of the mice
**PULMONARY DISEASE**					
Active anti-MPO autoimmunity with human neutrophil lysosomal extract infusion (84)	Brown Norway rats	++ to +++	14 days	Chronic inflammation and fibrosis seen at 14 days	Granuloma formation unusual in MPO-AAV
Active anti-MPO autoimmunity with localized single lung human neutrophil lysosomal extract infusion (83)	Brown Norway rats	++	10 days	Local and systemic effects of neutrophil degranulation	Infusion caused pulmonary damage in the absence of MPO-ANCA
Passive transfer of human PR3-ANCA into rats (123)	Wistar rats	++ to +++	24 h	*In vivo* evidence of pathogenicity of PR3-ANCA	Not strictly autoimmune
Perfusion of isolated rat lungs with primed human neutrophils and monoclonal PR3 Ab (124)	CD (SD) rats	++	3 h	Acute lung injury caused by neutrophil degranulation and free oxygen radicals	*Ex vivo* model Does not model the process of neutrophil migration to the lungs *in vivo*
**OTHER MODELS**					
Passive transfer of LAMP-2 Ab (125)	WKY rats	+ to ++	5 days	LAMP-2 is an additional target of ANCA	Not all Ab preparations are pathogenic
Immunization with FimH (125)	WKY rats	++	39 days	Molecular mimicry may underpin loss of tolerance to LAMP-2	Antigen processing not taken into account No clear demarcation between induction of immunity and effector responses
Spontaneous crescent formation in autoimmune-prone mice (126)	SCG/Kj mice	+++	Life span 120–135 days	Early onset severe disease	Derived from lupus prone strains Other auto-Ab present Significant immune complex deposition in glomeruli
Passive transfer of NET-loaded DC (59)	BALB/c and C57BL/6 mice	++ to +++	3 months	NETs may be involved in development of autoimmunity to MPO and PR3	Production of other auto-Ab in addition to ANCA Long model, requires multiple DC infusions
Passive transfer of PTU-induced abnormal NETs, PTU-induced MPO-ANCA production (127)	WKY rats	+	30 days	Prolonged MPO exposure via NETs may participate in loss of tolerance	Mild disease
Nephrotoxic serum nephritis (128)	C57BL/6 mice	+++	7–21 days	Mechanisms of severe nephritis	Mechanistically different effectors No induction of responses to ANCA antigens or transfer of specific cells or Ab

a *+, mild; ++, moderate; +++ severe*.

b *Only initial contribution listed due to space limitations*.

*Ab, antibody; ANCA, anti-neutrophil cytoplasmic antibodies; CD (SD) Cesarean derived (Sprague-Dawley); DC, dendritic cells; LAMP-2, lysosome-associated membrane protein 2; MPO, myeloperoxidase; NETs, neutrophil extracellular traps; NOD, non-obese diabetic; PR3, proteinase 3; PTU, propylthiouracil; Rag, recombination activating gene; SCG/Kj, spontaneous crescentic glomerulonephritis-forming/Kinjoh; SCID, severe combined immunodeficiency; WKY, Wistar Kyoto*.

In contrast to MPO-AAV, it has been difficult to use PR3-AAV models of disease to confirm unequivocally the pathogenicity of PR3-ANCA *in vivo*. Passive transfer of human PR3-ANCA from patients with active vasculitis into BALB/c wild type mice led to development of mouse ANCA. After some months many mice developed respiratory inflammation, and diffuse immunoglobulin deposition in the glomeruli, as opposed to the classical pauci-immune GN found in humans with PR3-AAV (114, 115). Subsequent studies passively transferred mouse anti-PR3 antibodies. PR3 antibodies raised in mPR3/neutrophil elastase (mNE) double-deficient mice and passively transferred to wild type mice were not able to induce vasculitis (116). Similarly, anti-PR3 antibodies from rats immunized with chimeric human/mouse PR3 were transferred into wild type mice. Despite high titres of PR3-ANCA, the mice did not develop clinical or histological features of vasculitis (117).

The presence of PR3-ANCA in itself did not cause disease in non-obese diabetic (NOD) mice, which develop spontaneous autoimmune disease. However, splenocyte transfer from these mice into NOD-severe combined immunodeficiency (NOD-SCID) mice caused crescentic GN and death. In contrast, splenocytes transferred into *Rag1*^−/−^ mice did not develop vasculitis. These experiments imply a significant role for the regulatory immune response in maintaining tolerance and limiting effector responses in PR3-AAV (118).

To overcome species-specific differences in PR3 structure and of Fc receptors, Little et al. transferred PR3-ANCA into mice with a humanized immune system. Irradiated NOD-SCID-*Il2r*γ^−/−^ mice were immune reconstituted with human hematopoietic stem cells. Passive transfer of human PR3-ANCA caused pauci-immune proliferative GN, and histological evidence of pulmonary vasculitis (119), providing the strongest *in vivo* experimental evidence to date for the pathogenicity of PR3-ANCA.

The latest attempts to overcome species differences and develop a representative murine model of PR3-AAV have involved mice that express human PR3. Mice with podocytes that express human PR3 (under a podocin promoter) did not develop disease after injection with anti-PR3 antibodies (120), perhaps due to a lack of access of the antibodies to the antigen that was expressed extravascularly on podocytes. A double-transgenic approach produced mice with human PR3 in neutrophils along with its co-receptor CD177. Vasculitis could not be induced through passive transfer of anti-PR3 antibodies, possibly because the mice may have been unable to process human pro-PR3 into mature PR3 (121). A third transgenic approach by Martin et al. generated mice expressing the mature form of human PR3, which appeared to be enzymatically active. In a model of zymosan-induced peritonitis, the presence of the hPR3 transgene increased neutrophil accumulation and enhanced neutrophil survival compared to PR3 wild type controls (122). Studies in experimental AAV using this mouse are yet to be reported.

## Other Models of AAV

These models are summarized in Table 2.

### Models of ANCA-Associated Pulmonary Vasculitis

Most animal models of AAV use glomerular disease as the primary endpoint. However, several animal models of pulmonary disease have been designed. Focal pulmonary hemorrhage and granuloma formation were identified in MPO-immunized Brown Norway rats 7 and 14 days after intravenous infusion of human neutrophil lysosomal extract and hydrogen peroxide (84). These changes were accompanied by haemorrhagic lesions within the intestines. Of note, granuloma formation is more closely associated with PR3-AAV, rather than MPO-AAV. A variation of this model explored the role of ANCA in the lung (83). Isolated left lungs of MPO-immunized rats were perfused with a neutrophil lysosomal extract. The isolated left lung displayed inflammatory lesions in both immunized and non-immunized mice, though more extensive in the immunized group; in contrast, there were inflammatory infiltrates also in the right lung only in the presence of anti-MPO antibodies.

Other systems have modeled PR3-AAV associated pulmonary involvement. C-ANCA from patients with GPA was transferred to Wistar rats (123). All animals displayed marked pulmonary vasculitis 24 h after antibody transfer in a dose-dependent manner [despite the limited homology between human and mouse PR3 (106)], with no disease observed in mice treated with control IgG. Co-perfusion of isolated rat lungs with primed human neutrophils along with murine monoclonal PR3 antibody rapidly caused oedema formation and increased microvascular permeability (124). This was not observed after perfusion of primed neutrophils alone or anti-PR3 antibodies alone, suggesting synergistic roles for anti-PR3 antibodies and neutrophils in pulmonary pathology in AAV.

Pulmonary findings have been described in models that otherwise focus on renal disease. Pulmonary capillaritis developed in 5 of 16 mice after passive transfer of high doses of anti-MPO splenocytes into *Rag2*^−/−^ mice, and in 2 of 6 wild type mice after transfer of anti-MPO antibodies (15). A similar result was also seen in MPO-immunized *Mpo*^−/−^ mice which were irradiated and reconstituted with bone marrow from wild type mice (25), and after transfer of human PR3-ANCA into irradiated NOD-SCID-*Il2r*γ^−/−^ mice reconstituted with human hematopoietic stem cells (119). Pulmonary lesions were occasionally and inconsistently detected after transfer of MPO peptide-specific CD4+ T cell clones, despite all mice developing glomerulonephritis (18).

### Experimental AAV Induced by Autoimmunity to LAMP-2

Whilst MPO and PR3 are the most common antigenic targets of ANCA, other neutrophil proteins have been identified, including lysosome-associated membrane protein-2 (LAMP-2). Autoantibodies to LAMP-2 have been detected in humans, and were detectable in WKY rats after immunization with LAMP-2; these antibodies induced acute focal necrotising GN on transfer (125). The bacterial adhesin FimH contains a sequence with strong homology to the immunogenic epitope of LAMP-2 (with eight of the nine amino acids identical). Immunization with FimH induced anti-LAMP-2 antibodies and pauci-immune GN. It was proposed that autoimmunity to LAMP-2 was due to molecular mimicry, potentially explaining the temporal relationship between bacterial infection and AAV. The authors hypothesized that antibodies to LAMP-2 may alter presentation by neutrophils of cytoplasmic antigens, facilitating generation of autoantibodies to MPO or PR3. The results of these animal experiments were not replicated by another research group (129), though anti-LAMP-2 antibodies were found in European AAV cohorts (130, 131).

### ANCA and Disease in Lupus Prone Mice

Clinically, ANCA can be detected in some patients with systemic lupus erythematosus (SLE) (132, 133), and recent observations suggest that this is associated with a more vasculitic renal phenotype (134). Similarly, some mouse strains that develop systemic autoimmunity also have perinuclear ANCA formation and crescentic GN. A proportion of lupus-prone MRL/*lpr*^*fas*^ mice develop MPO-ANCA that bind to MPO on the neutrophil surface. These mice exhibit a phenotype with some characteristics of AAV (135, 136). MRL/*lpr* mice have been further genetically modified to knockout *Nos3*, in order to investigate the potential for endothelial nitric oxide synthase (eNOS, or NOS3) to inhibit vascular inflammation. Paradoxically, NOS3 deficient mice had accelerated onset and increased incidence of renal vasculitis compared with control MRL/*lpr* mice (137).

Derived from two lupus prone strains, MRP/*lpr* and BXSB, and selectively bred for crescent formation, spontaneous crescentic GN forming/Kinjoh (SCG/Kj) mice have more extensive glomerular crescents in all mice (126), though the renal disease may be largely immune complex mediated (138), with development of disease and progression to renal failure significantly delayed by calorie restriction (139). Initial suggestions that neutrophils are required for ANCA-associated GN development were based on experiments in these mice, in which increased peripheral neutrophil numbers and glomerular neutrophil infiltration correlated with disease (140). Multiple genetic associations with MPO-ANCA development in SCG/Kj mice have been defined (141). This model has also been used to investigate possible treatments for ANCA-associated GN, including 15-deoxyspergualin (142, 143) and omega-3 fatty acid eicosapentaenoic acid (EPA) (144).

Dendritic cell transfer studies have implicated NETs in the development of autoimmunity to MPO and PR3 (59). NETosis-prone neutrophils from naïve mice were co-cultured with myeloid dendritic cells (mDC), resulting in mDC uploading with NET components including MPO. Wild type mice subsequently immunized with NET-loaded dendritic cells (but not dendritic cells exposed to apoptotic or DNAse1 treated neutrophils), lost tolerance to MPO and PR3 and produced ANCA. Furthermore, induction of ANCA was associated with moderate to severe renal injury. However, antibodies to other targets were also produced, including single-stranded and double-stranded DNA, implying a more generalized loss of tolerance consistent with SLE or an SLE-like syndrome.

It is difficult to isolate the effects of anti-MPO and anti-PR3 autoreactivity in these models. While ANCA are present, so are other autoantibodies, including anti-double stranded DNA (dsDNA) antibodies. These models are potentially useful as models of vasculitis occurring in the context of SLE with concurrent MPO-ANCA, where in humans segmental necrosis is more likely and in which ANCA may play a role (134).

### Drug-Induced Experimental AAV

The development of MPO-ANCA and clinical features of AAV has been reported in association with many different drugs [review in Gao and Zhao (145)]. The anti-thyroid drug propylthiouracil (PTU) is associated with production of MPO-ANCA in up to 30% of patients, with some patients developing MPO-AAV (146). *In vitro*, incubation of human neutrophils with PTU in the presence of phorbol myristate acetate (PMA) induces NETs. These PTU induced NETs have an abnormal conformation and are more resistant to breakdown by DNase I. It is thought that prolonged MPO exposure on NETs participates in loss of tolerance to MPO, and subsequent ANCA production. Passive transfer of these abnormal NETs into WKY rats caused development of MPO-ANCA and pulmonary capillaritis. In an active model of disease, MPO-ANCA and features of vasculitis developed in rats given PTU in addition to intraperitoneal PMA, with evidence of DNase1-resistant abnormal NET formation. In contrast, mice given only intraperitoneal PMA developed NETs without development of MPO-AAV (127). In a similar model of PTU-induced MPO-ANCA associated vasculitis, BALB/c mice were given PTU; NET formation was attenuated by peptidylarginine deiminase (PAD) inhibition, with lower MPO-ANCA titres (147).

### Autologous Phase Anti-GBM GN (Nephrotoxic Serum Nephritis)

The most commonly used model of severe and rapidly progressive GN is the autologous phase (accelerated or non-accelerated) “anti-GBM” model, also known as nephrotoxic serum nephritis (128). This model has been used as proxy for ANCA-associated GN (148). However, while glomerular injury may appear similar histologically, the pathogenesis of this model is substantially different to that of AAV. Glomerular injury in this model is not due to autoimmunity and autoreactivity to MPO is not present (149, 150).

The relative contributions of cellular and humoral effectors in this model depend on the strain and species of rodent used, and the nature, timing and dose of the foreign globulin. The initial phase of injury is neutrophil mediated, though unlike ANCA-associated vasculitis, here the heterologous globulin binds to the glomerulus and neutrophils are retained in glomeruli via this *in situ* immune complex mediated disease (89, 151). Subsequently, in non-accelerated iterations of this model, immunity to the foreign globulin (usually raised in sheep or rabbits) as a foreign antigen (not as an autoantigen) develops. Anti-sheep (rabbit) antibodies and/or anti-sheep (rabbit) T cells localize to glomeruli as the antigen (the heterologous globulin) is bound to the glomerulus (152, 153). In the accelerated model, immunity to sheep (rabbit) globulin has been induced by priming with the foreign antigen in adjuvant, but injury does not occur until the antigen localizes to the glomerular basement membrane.

Thus, while there may be some similarities in cellular effectors, critical differences in the induction of immunity (including regulatory T cells), and effector mechanisms mean that this model does not represent ANCA-associated GN. It should not be described as autoimmune and care should be taken in extrapolating results in these models to autoimmune ANCA-associated GN.

## Discussion and Concluding Remarks

The use of animal models of AAV, especially combined with careful observational and *in vitro* human studies has been instrumental in the major advances in our understanding of the pathogenesis of AAV, ranging from the pathogenicity of ANCA, through to elements of loss of tolerance, the role of infection and the participation of cellular immunity. Animal studies on the functional role of complement have led to human trials of new therapies based on complement inhibition. Each of the models used in this search and described in this review has informed us about different aspects of pathogenesis. Table 3 summarizes insights into the pathogenesis of AAV obtained by using animal models and their translational potential.

**Table 3:** Influence of animal models on selected elements of the pathogenesis of ANCA-associated vasculitis.

**Observation from humans and *in vitro***	***In vivo* animal models**	**Future directions**
**RISK FACTORS**		
Incidence increases with age (1)	Anti-MPO antibodies transferred into aged mice associated with more severe disease (71)	PR3 models of disease
PR3-AAV significantly associated with HLA-DP4 (154) and HLA-DR15 (155)		Understanding mechanism of risk
**LOSS OF TOLERANCE**		
Association between AAV and infection (102) Autoimmunity to PR3 may be triggered by exposure to complementary proteins (156)	Anti-MPO immunity triggered by exposure to bacterial peptide with MPO sequence homology (104) Autoimmunity to LAMP-2 developed after immunization with a homologous peptide from FimH (125)	Re-induction of tolerance
**PATHOGENICITY OF ANCA**		
Presence of ANCA in patients with AAV (11) *In vitro*, capacity of ANCA to induce neutrophil stimulation and degranulation (12, 34) Treatment response to autoantibody and B cell depletion	Passive transfer anti anti-MPO antibodies caused development of GN (15) Binding of ANCA to neutrophils induces glomerular leukocyte adhesion (53)	Clarify the role of autoantibody depletion in induction of disease remission Role for treatments which alter the antibody itself
**NEUTROPHILS AS EFFECTORS**		
Paucity of immunoglobulin in renal biopsies suggests antibody-independent mechanisms Number of activated neutrophils in glomeruli is associated with severity of renal disease (13) *In vitro*, neutrophil degranulation after stimulation with ANCA causes endothelial damage (157)	Presence of ANCA-like antibodies without neutrophil activation is insufficient to cause disease (24) Increased peripheral neutrophil numbers and glomerular neutrophil infiltration correlated with disease (140) Neutrophil depletion protects from disease (35) G-CSF administration exacerbates renal injury (31)	Potential therapies for AAV that de-activate neutrophils
**EFFECT OF CYTOKINES AND CHEMOKINES ON NEUTROPHIL PRIMING, MIGRATION AND ADHESION**		
Association between AAV and infection (102, 158) *In vitro*, priming of neutrophils is required for optimum activation by ANCA (159)	Passive transfer of anti-MPO antibodies in conjunction with LPS (neutrophil priming) causes more severe disease (32) Engagement of TLR4 on glomerular endothelial cells with highly-purified LPS associated with production of CXCL1 and CXCL2, leading to increased neutrophil migration, adhesion and transmigration (47) Upregulation of β2 integrins, mediated by TNF, is associated with decreased leukocyte rolling and enhanced adhesion (50)	Further studies of anti-TNF therapies as potential treatmentStudies of inhibiting neutrophil adhesion in AAV
**COMPLEMENT IN AAV**		
Role of complement first elucidated in animal models, and then confirmed in humansPatients with AAV have increase plasma levels of alternative pathway activation markers (160)	C3 depletion prevents GN in mice in passive transfer model (17) Plays a role in neutrophil retention within the glomerulus and subsequent glomerular injury (22)	Role of complement inhibition in disease management e.g. CCX168/avacopan
**NEUTROPHIL EXTRACELLULAR TRAPS (NETs)**		
NETs at sites of vascular injury (55)	After activation by ANCA, neutrophils undergo cell death and develop NETs, which promotes autoimmunity to MPO and propagates glomerular endothelial damage (161)	Role for enhanced NET degeneration for disease treatment
**ROLE OF T CELLS**		
Tubulointerstitial and intraglomerular T cells associated with worse renal injury (55)	Induction of T cell autoimmunity to MPO results in NCGN, even in the absence of B cells and ANCA (16) T cells involved in neutrophil chemoattraction through production of IL-17A (80) Depletion of peripheral regulatory T cells associated with more severe disease (93)	Understanding the mechanism of CD8 T cell mediated end-organ damageRegulatory T cell based therapies for AAV
**MONOCYTES**		
Activated by ANCA (10) Presence of monocytes and macrophages in renal biopsies Soluble CD163 in urine, which is shed by monocytes, is strongly associated with active renal vasculitis (66)	Monocyte depletion reduces glomerular necrosis and crescent formation after passive transfer (9)	Pre-clinical evaluation of monocyte-related biomarkers and therapies

*AAV, ANCA-associated vasculitis; ANCA, anti-neutrophil cytoplasmic antibodies; G-CSF, granulocyte colony stimulating factor; GN, glomerulonephritis; HLA, human leukocyte antigen; LAMP-2, lysosome-associated membrane protein-2; LPS, lipopolysaccharide; MPO, myeloperoxidase; NCGN, necrotizing crescentic glomerulonephritis; NETs, neutrophil extracellular traps; PR3, proteinase 3; TLR4, toll-like receptor 4; TNF, tumor necrosis factor*.

However, the development of a single, accurate and translatable animal model of ANCA-associated vasculitis has challenged researchers for decades. This disease, and its pathogenesis, is unique in multiple respects. Firstly, it is a systemic autoimmune disease, though there is only one autoantibody clinically detected. ANCAs themselves are unusual in that they cause activation, rather than destruction of the target cell. Effector responses are complex. The autoantigens themselves are both interesting and unusual; whilst MPO and PR3 are present systemically, the disease manifestations are in organs where the autoantigen is not expressed. Clinically, the disease is heterogeneous, with significant variability in genetic predisposition, environmental risk factors, severity, organ involvement, and risk of relapse.

Given the multi-faceted pathophysiology of AAV, it is not surprising that no single model can recapitulate all aspects of disease. Several disease models are required to comprehensively model and study AAV. Thus far, passive transfer models have proven valuable in studying early effector responses and have resulted in the translation of anti-C5aR therapies into Phase 2 and Phase 3 clinical trials (21, 42, 162). Further work is necessary to refine and establish animal models that reflect human disease as accurately as possible. Furthermore, the models should be reliably reproducible, tractable and transferrable between laboratories, to promote collaborative research and treatment development. Establishment of such models will facilitate a human-rodent-human iterative approach which may accelerate understanding, discovery and research translation.

Despite the extensive advancement in knowledge over the past decades, treatment of ANCA-associated vasculitis remains non-specific and toxic. With the likely exception of rituximab, new therapies have not been more efficacious than standard of care, itself associated with significant risks of infection and malignancy. Given the complexity of the pathophysiology, treatment may need to be multi-targeted, requiring collaborative research for development and testing.

Given the emerging knowledge of the differences between PR3-AAV and MPO-AAV, there is a growing need for a model of PR3-AAV. Much can be learnt from the experiences of previous attempts to develop an animal model. The ideal model would likely require human mature PR3 expression in the neutrophil through genetic mutation, manipulation of neutrophil numbers and PR3 membrane expression, as well as consider the importance of Fcγ receptors in the ability of ANCA to activate neutrophils. Furthermore, mice transgenic for human immune genes, such as HLA, may be used for understanding the strong genetic associations identified with PR3 (154, 155, 163, 164) and model key pathways in loss of tolerance and effector responses. Currently there are no published models of eosinophilic granulomatosis with polyangiitis (EGPA). A significant proportion of people with EGPA have MPO-ANCA antibodies and recent GWAS studies suggest a combination of genes relevant both to autoimmunity and to allergy/eosinophil function may be involved in EGPA (165).

In the future, the current animal models need to continue to evolve to address key clinical questions at hand. Examples of how these questions might be addressed are outlined below. ANCA associated vasculitis is largely a disease of older people, and older age is associated with worse renal outcomes and increased mortality, with more complications of treatment (166). The use of aged mice in translational research is increasing, and allows a unique opportunity to more closely mimic human disease (167). Multiple genetic associations with AAV have been identified, especially with regards to antigen presentation (154, 155, 168), which could be mechanistically explored in HLA transgenic mice. The role of concurrent infections, including but not limited to latent cytomegalovirus infection, in loss of tolerance to ANCA antigens, as well as disease outcomes, needs to be considered (169, 170).

At this stage, animal models of MPO-AAV are unable to meaningfully mimic chronic end-organ disease. Clinically, how to best manage AAV in the medium to long term, given its chronic relapsing autoimmunity with tissue injury and damage is a major challenge, and can lead to use of long-term immunosuppression that may or may not be required. Precision medicine would ideally include the capacity to recognize patients at risk of relapse, and reliably identify relapses before end-organ damage ensues. Furthermore, as in many chronic inflammatory diseases, treatments that prevent progressive fibrosis are needed to preserve function after tissue damage mediated by anti-PR3 and anti-MPO autoimmunity.

## Author Contributions

LS and AK conducted literature searches, selected relevant articles, planned the format of the article, and wrote the article. LS, SH, and AK reviewed, edited, and finalized the article for submission.

### Conflict of Interest

The authors declare that the research was conducted in the absence of any commercial or financial relationships that could be construed as a potential conflict of interest.
